# Is Generation Z Ready to Engage in Entomophagy? A Segmentation Analysis Study

**DOI:** 10.3390/nu15030525

**Published:** 2023-01-19

**Authors:** Irene (Eirini) Kamenidou, Spyridon Mamalis, Stergios Gkitsas, Ifigeneia Mylona, Aikaterini Stavrianea

**Affiliations:** 1 Department of Management Science and Technology, School of Business and Economics, International Hellenic University, 65404 Kavala, Greece; 2 Department of Sciences & New Technologies, School of Primary Education, Aristotle University of Thessaloniki, 54124 Thessaloniki, Greece; 3 Department of Communication and Media Studies, National and Kapodistrian University of Athens, 10559 Athens, Greece

**Keywords:** entomophagy, Generation Z cohort, consumer attitudes, consumer behavior, consumer segmentation, marketing communication

## Abstract

This study examines the behavior and attitudes of adult Generation Z cohort members in relation to entomophagy. Specifically, it explores their familiarity with insect consumption, prior experience, and willingness to consume certain insect-based foods and drinks. Lastly, the Z cohort is segmented based on their behavior and attitudes. Through online quantitative research, a valid sample of 742 questionnaires was collected. Data analysis included descriptive statistics, reliability analysis, factor, hierarchical cluster, and K-means cluster analysis, as well as chi-square tests. Results revealed that 41.4% are familiar with what insect consumption is, and no one had previously engaged in entomophagy. The insect-based food that the Z cohort is most willing to try is bakery products containing insect flour. The 88.5% of the Z cohort is not willing to replace meat protein with insect protein, and 20.4% are interested in obtaining more information about entomophagy. Moreover, 6.3% of the Z cohort is “willing” to participate in sensory tests, but when contact information was requested, only one factual name with phone number was provided. Segmentation of the Z cohort’s behavior was performed based on eight variables and four segments were identified: the “Future potential insect consumers” (29.1%), the “Rejecters” (26.7%), the “Disgusted, prefer to starve” (22.2%), and the “Inconsistent” (22.0%). Overall, the Z cohort is not food neophobic, but is unwilling to engage in entomophagy. Communication strategies are suggested to increase awareness and provide information about entomophagy and its benefits.

## 1. Introduction

The daily intake of dietary proteins is crucial for human nutrition, as they are important for biological functions such as tissue building [1]. Protein is also the most satiating of the macronutrients, since a diet rich in protein produces a feeling of satiety [2]. In recent years, the demand for animal protein has increased rapidly due to population growth, income increase, and urbanization [3,4].

High consumption of animal protein, i.e., meat consumption, has been linked to health problems such as cancer, cardiovascular disease, and type 2 diabetes [5]. Furthermore, it is estimated that the livestock sector itself contributes to 14.5% of total anthropogenic greenhouse gas (GHG) emissions worldwide [6].

The importance of protein intake is undisputed. However, the problems that animal protein consumption poses to the sustainable future of the planet and to human health are leading to the consideration of alternative sources of protein intake. One of the alternative sources of protein intake, which is gaining more and more interest, is the consumption of insects [7,8].

Insects are mainly composed of protein and fat, followed by fiber, ash, and nitrogen-free extract [9]. The protein content of insects is comparable to that of conventional meat such as pork and beef, and they contain several essential amino acids [10,11]. Specifically, insect proteins have a high content of most essential amino acids, especially lysine and threonine, and a low content of methionine and cysteine [12]. The quality of amino acid composition of insects from the orders Lepidoptera, Orthoptera, Hymenoptera, and Coleoptera meets or exceeds current adult requirements on average, with levels comparable to milk, beef, eggs, and soy [13]. For example, the mulberry silkworm consumed in Thailand contains more than 40% of essential amino acids, and has a ratio of 0.6 between essential and nonessential amino acids [14]. In addition, the black soldier fly is rich in aromatic amino acids, which are nearly three times the FAO/WHO requirement for older children, adolescents, and adults (4.10/100 g) [15]. The fat content of insects can be up to 70% in polyunsaturated fatty acids, while ruminant milk contains about 30% in comparison [16,17]. Insects are also a rich source of minerals and vitamins [18,19]. For example, most food insects contain more iron and calcium than conventional meat [9,10]. In addition, health benefits have been attributed to insect consumption, such as a significant improvement in gut health and a reduction in systemic inflammation [20].

Insect consumption (entomophagy) is not a new concept, deriving solely from population growth and global sustainability issues. Entomophagy is recorded as far back as the writings of the Old Testament, the 4th century BC in ancient Greece, and the 1st century AC of Romans [21]. In many parts of Latin America, Africa, Australia, and Asia, insects like grasshoppers, locusts, ants, termites, caterpillars, and beetles are consumed by native or local populations [10]. It is estimated that insect consumption is part of the diet for at least 2 billion people [22], including some developed countries like Japan [23], where, for example, Vespula spp. (wasps) are consumed [24]. Concerning Western civilization, the suggestion that insects could be used as a food source for people backdates to the 19th and 20th centuries [25].

While entomophagy has many benefits, intolerant reactions and allergies have been recorded, for instance in studies of allergic reactions in countries where insect consumption is traditional [26,27]. In the Western world, some studies have found allergies caused by entomophagy, either through cross-reactivity with allergies to dust mites and crustaceans [28,29], or through primary sensitization [30]. On the other hand, De Gier and Verhoeckx [31] reported that adverse reactions after insect consumption are rare. The European Food Safety Authority (EFSA) [32] concluded that “the consumption of the evaluated insect proteins may potentially lead to allergic reactions. It may particularly be the case in subjects with pre-existing allergies to crustaceans, dust mites and in some cases molluscs”. The same source acknowledges that allergic reactions towards food represent 2–9% of the population, depending on the food type.

The growing concern for food sufficiency [33] and the sustainable future of the planet, combined with the demonstrated high protein value of edible insects, has sparked interest among researchers in the acceptance of insect consumption in other parts of the developed world [19,34,35,36,37,38]. However, preceding studies show that entomophagy is considered a food taboo in Western cultures [39,40]. In countries where entomophagy is not practiced and acceptance is low, prior research reveals that the main reasons are consumer disgust with insects as food [22,41,42,43], food neophobia [41,44,45], perceived health risk [46,47], and negative attitudes [45,48].

Disgust has been identified in the literature as the most important predictor of consumer rejection of insect consumption [43,49,50,51,52,53]. Hamerman [53] (p.320) states that disgust is “a type of food rejection motivated by “offensive properties” that lead to the presumption that the food itself will have an unpleasant taste or make people sick”. Disgust with eating insects results also from the perception that insects may be carriers of disease, and thus food contaminants [54]. The response to food (e.g., disgust), is of particular interest because it is triggered by both distal (visual, auditory, orthonasal olfaction) and proximal (gustatory, retro nasal olfaction, tactile) sensations [55]. While many factors play a role in consumer acceptance, food neophobia has also been identified as a major barrier to the inclusion of insects in the human diet [41,43,44,56]. Food neophobia largely determines consumers’ decision-making when they must choose between unfamiliar new foods and those that are already familiar [57].

Although behavior related to insect consumption has been studied and is steadily gaining interest, There is a lack of studies of studies that focus on generational cohorts and insect consumption behavior (from all perspectives). To our knowledge, seven studies exist [47,58,59,60,61,62,63], one of which is a bachelor thesis [61], and one with only a single question about entomophagy [62].

According to the generational cohort theory, generational cohorts include people who are born in a specific place during a specific time period and have experienced the same life-changing events at their “coming of age” that shape the behavior of the respective cohort [64,65,66]. In this regard, it is important to note that generational cohorts and generations are two different concepts, the latter referring only to a year span of 20–25 years [66,67].

The study of consumer patterns based on their generational cohort is a topic that has received increasing attention in the food marketing and general marketing field [68,69,70,71], and is under-researched compared to other consumer characteristics. As Chaney et al. [72] note, the concept of generational cohorts has been studied extensively in the social sciences, while it has only been studied at the borderline in the marketing field. Moreover, when studying consumer characteristics, generational cohorts are considered by marketers to be a more significant indicator than age [73].

The Z generation cohort is currently the youngest cohort of adults, and includes individuals born from 1995 to 2009 [74]. As a generation growing up in the face of climate change, they tend to be more aware of sustainability issues, although as digitally savvy consumers they also value aspects such as convenience and innovation [62,75,76]. Members of this cohort are highly educated and creative [76]. They are tech-savvy, believing that the Internet, social media, and smartphones (which are also considered a status symbol), are very important [77]. They believe that online information is accurate and therefore use web search engines [78]. Overall, they have been familiar with technology throughout their lives as they were born in the digital world [76,79]. They do not tend to be brand loyal, and are experience motivated [76,80]. Therefore, in their digital world, extended, virtual, augmented, and mixed reality, among others, provide them with a user experience [81], consequently enhancing consumers’ experiential quality [82]. Moreover, they exhibit a high level of individualism, which is mainly due to the fact that they have grown up in smaller families compared to previous cohorts [83]. They also live longer in their parents’ homes compared to previous cohorts [84], and they are traditional and responsible [74]. On the other hand, they have great spending power, and often buy products related to their personal brand and invest a lot of time in creating networks via social media [83]. However, it is important to note that the beliefs and behaviors of this cohort are not yet fully formed, as global and domestic events at their coming of age continue to occur [85].

Prior studies focusing on the Z cohort are emerging in different domains of academia, such as workforce, food consumption, retailing, tourism, and engineering, e.g., [65,76,81,86,87,88]. Studies on insect consumption that focus on or include the Generation Z cohort are few [47,58,60,62] to the authors’ knowledge, indicating the need for further research. Therefore, this study attempts to partially fill this academic research gap by investigating and recording the Z cohort’s behavior and attitudes towards entomophagy, and segmenting participants according to them.

With the abovementioned background at hand and focusing on the Generation Z cohort regarding the consumption of insects, the following research questions arose:

- RQ1: Is the Generation Z cohort familiar with what entomophagy is?
- RQ2: Has the Generation Z cohort any previous experience with edible insect consumption?
- RQ3: Are the members of the Z cohort willing to try certain edible insects or insect-based foods and drinks?
- RQ4: Are the members of this cohort willing to replace animal protein in their diet with insect protein?
- RQ5: Is the Generation Z cohort interested in learning more about entomophagy and in participating in sensory testing of insect-based foods?
- RQ6: Can the Z generation cohort be grouped based on their behavior and attitudes towards consuming insects and insect-based foods?

This research draws data from Greece, a European country where Greek households have suffered income losses since 2009 until today due to the 12-year recession, the lockdowns, and business closures in the Covid era, as well as the problems resulting from the side effects of the increase in energy prices due to the Russian–Ukrainian war. In particular, the older members of the Greek Z cohort have witnessed family members losing their jobs due to the economic crisis (and Covid and its aftermath). Specifically, Stavrianea and Kamenidou [89] found that during the financial crisis, 35.7% of the Greek Z cohort witnessed at least one family member losing their job, while Kamenidou et al. [90] found that households experienced food security problems, which led them to produce their own food and to stockpile where possible. Additionally, Anastasiadou et al. [91] found that during the pandemic, Greek consumers presented high uncertainty and stockpiled essential goods to feel secure.

In answering the above research questions, the research gaps that the study identified and addresses, and thus its added value to academia, are the following. Firstly, generational cohorts in consumer behavior research, even though an emerging topic in several domains e.g., [76,81,86] is still quite limited, and therefore additional research is required. Secondly, it focuses on the Generation Z cohort, which is a rising topic in the food marketing field [68,69,92]. As Priporas et al. [76] (p. 1) state, “The biggest future challenge for marketing and consequently for retailing seems to be Generation Z”. Thirdly, it focuses on the Generation Z cohort and their consumer behavior towards entomophagy, where there is a dearth of studies, as only four studies (in total) were found. This raises the need for further investigation. Fourthly, it segments the Z cohort based on behavior and attitudes towards entomophagy, a subject that lacks research. Only few studies to our knowledge deal with consumer segmentation and entomophagy [46,93,94], none of which incorporate generational cohorts. Lastly, it provides an in-depth understanding of the behavior and attitudes of the Greek Z cohort towards entomophagy. This knowledge therefore predicts their future acceptance and trends towards the consumption of edible insects and insect-based food and drinks. Therefore, this study contributes to the abovementioned from the theoretical point of view to academia. It is worth mentioning that regarding the Greek context and entomophagy, there is only one study from the customers’ point of view, that of Giotis and Drichoutis [95], who investigated the acceptance and willingness to pay for direct and indirect entomophagy.

## 2. Materials and Methods

A multistage approach has been utilized for this study. Initially, an extensive literature review provided an overview of insect consumption, and presented the main axes of the research questions and components of the questionnaire. It also offered insight into young peoples’ and generational cohorts’ behavior and attitudes in relation to insect consumption, the latter being the focus of the study. In this way, the first draft of the questionnaire was developed, and its content validity was ensured by adopting items used in previous research [96]. In the following second stage, a qualitative research approach was adopted through an informal discussion [97] on entomophagy. For this to be feasible, 58 university students, all members of the Generation Z cohort were recruited. The main reason for this procedure was to validate the questions of the questionnaire and to add new variables if needed. From this procedure, attitude measurement questions (12 items) were added in the final questionnaire. In the third stage, the revised version of the questionnaire was pilot tested to identify any comprehension and wording problems, thus establishing face validity [96]. For this procedure, data was collected two ways: online within three days (72 participants), and via an aided self-administered questionnaire [98] with 41 students during class. The participants’ answers in the pilot test were excluded from the final sample. It is noted that the first question of the research instrument was the consent of the participants to use the data for analysis according to GDPR law (law 4624/2019), and the last referred to comprehension issues, or other issues that were not detected during its development. Based on the suggestions of the participants, changes were applied (mainly wording issues). In the fourth and last stage, the quantitative research was carried out. The final version of the questionnaire was distributed online through a link that allowed responses for 34 days (28 October to 30 November 2022). The questionnaire was answered initially by university students (convenience sample). They were then invited to forward it to their friends and acquaintances, who were members of the Generation Z cohort, to fill it out (snowball sampling). In order to contribute to the research, criteria were applied. Specifically, the participant had to be Greek, or living in Greece from at least his/her 16 years of age (in order to share the same events in their coming of age), was an adult member of the Greek Generation Z cohort, i.e., born between 1995–2004 (to be at least 18 years old in 2022, the year of the research), and have access to the internet. In this way, 742 questionnaires usable for the analysis were obtained. Finally, it should be noted that the final version of the questionnaire included a question about consent and the confidentiality statement [99]. The obtained sample was considered sufficient in terms of size and the statistical analysis performed [100,101].

Regarding the questionnaire, Table 1 lists the questions used in the study, the authors from whom they were adopted, and the type of scale. The scale used was adopted from the authors, or else stated otherwise with an *. The 7-point Likert scale had the following choices: “1 = Completely disagree, up to, 7 = Completely agree, with 4 = the neutral point, i.e., neither disagree nor agree.” The Likert type scale had the following answers: “1 = Completely/totally unlikely, up to, 7 = Completely/totally likely, with 4 = the neutral point, i.e., neither likely nor unlikely” (for the intention to engage in entomophagy and the perceived risk of entomophagy to human health). As for the Cicatiello et al. [42] scale, the initial scale was a Likert type scale, which was modified to a Likert scale based on the suggestions and requests of participants in the pilot test.

Data analysis with the program SPSS ver. 28 included descriptive statistics, reliability and factor analysis, hierarchical cluster, and K-Means cluster analysis, as well as chi-square tests. The latter was used to explore the segments profile.

## 3. Results

### 3.1. Sample Profile

Male subjects were overrepresented (Table 2), as were younger members of the Z cohort. With reference to marital status, the overwhelming percentage of respondents were single, had secondary school education, and lived in the city. With respect to occupation, most of them were students, and concerning the net monthly family income, for most participants, it was ≤ EUR 1000.0. It should be noted that for members of the Greek Z cohort who live or study alone, their income was calculated as the amount they receive from their parents plus any other income they may have (e.g., from a part-time or full-time job).

### 3.2. Awareness and Behavior towards Entomophagy

Regarding their dietary habits, 43.5% of the Z cohort reported that they are meat eaters (they eat meat daily or several times a week), 45.0% reported that they follow a balanced/Mediterranean diet (they eat all types of foods, with an emphasis on vegetables, legumes, fruits, and fish, and do not eat meat very often per week). In addition, 7.0% were flexitarian and 4.5% had a pescatarian, vegetarian, or vegan diet. Regarding self-reported familiarity with insect consumption (RQ1), 41.4% (N = 307) responded that they have heard of it and know what it means; 41.8% (N = 310) have heard of it but do not know what it means; and 16.8% (N = 125) have never heard of eating insects. Regarding previous experience with entomophagy (RQ2), no one (0.0%) has ever eaten edible insects (to their knowledge).

Table 3 lists the insect or insect-based foods or drinks that participants were presented and asked to state if they are willing to try (RQ3; presented in descending percentage order of willingness to consume them). From the analysis, the participants are willing to try (mainly) those foods where the insects are not visible. Specifically, the Z cohort is most willing to try (top three) bakery products containing insect flour, rice/pasta enriched with insect flour, and fried/grilled/toasted whole insect. From the 22 items presented, only one item has a percentage >20.0% (bakery products containing insect flour). On the other hand, the Z cohort is least willing to try scarabs (91.5%), cockroaches (90.7%) and caterpillars (90.3%).

In following, participants were asked if they would be willing to replace animal protein with insect protein (RQ4). The overwhelming majority (88.5%, or 657 participants) answered no, whereas 11.5% (N = 85) replied yes. Additionally, when asked if they were interested in receiving more information on entomophagy (RQ5), 20.4% (N = 151) responded yes and 79.6% (N = 591) said no. Moreover, when asked if they would be willing to participate in sensory testing (RQ5), 47 participants (6.3%) replied yes, while 695 (93.7%) answered no. For those participants whose response was yes, there was an optional open-ended question asking for their contact information. Of the 47 participants, only 13 left a name (first or last) without a phone number, and only one male subject left a phone number and a name that was factual.

### 3.3. Segmentation of the Greek Z Generation Cohort

The segmentation variables that are considered in the analysis (RQ6), are those that researchers have demonstrated that influence intention to consume edible insects, namely: Food neophobia, disgust, perceived risk of consumption, barriers to entomophagy (extrinsic and perceived intrinsic cues), and attitudes toward insect consumption. Lastly, intention to consume was included.

For the questions/variables with multiple items, factor analysis with principal component analysis and varimax rotation was performed to reduce the number of items and make them more manageable for further analysis. Items with loadings on factor <0.4 or loading on more than one factor resulted in their removal [106].

Table 4 presents the dimensions/constructs (derived after factor analysis; eigen values >1 and eliminating items with loadings <0.4 or loading on more than one factor). Table 4 also displays the reliability of the overall scale and per construct. Reliability measured with Cronbach alpha (overall and per dimension) yielded a satisfactory result, ranging from 0.753–0.971 [107].

Regarding the attitude question, the items were adopted from the qualitative research, and factor analysis extracted three dimensions, named as follows: “Conspiracy theories”, “Consumption without knowledge” and “Starvation than entomophagy”. The names of the dimensions were based on the items that each dimension incorporated. For example, the item “Everything implies that with the new world order, in the future, few elites will control food distribution and for so, they are trying to make us believe that it is good to eat insects so that our subconscious will accept it” was included in the “Conspiracy theories”. The item “How many times have we eaten flies or other insects and do not know it? Now at least we will eat them knowing what we are eating” was included in the “Consumption without knowledge” dimension. Lastly, the item “I would rather starve to death than eat an insect” was an item in the “Starvation than entomophagy” dimension.

### 3.4. Cluster Analysis

As the above variables that were factor analyzed resulted to seven dimensions, all were utilized to group the Generation Z cohort according to their behavior and attitudes, adding one more variable (perceived health risks). Therefore, continuously, cluster analysis was applied based on the dimensions/variables: “Food neophobia”, “Barriers to entomophagy (extrinsic and perceived intrinsic cues)”, “Disgust”, “Perceived health risks”, “Conspiracy theories”, “Consumption without knowing’, “Starvation than entomophagy” and “Intention to consume/try”.

In order to find the best segmentation solution, data analysis first applied hierarchical cluster analysis (Ward’s method) to identify the solutions of segments and estimation of their centroids [100]. Then, K-means cluster analysis was practiced, and the potential solution was tested and compared with others obtained from random data subsets [108,109]. In addition, different numbers of clusters were examined, considering that the final extracted clusters should have a practical and physical meaning [110]. Lastly, the ANOVA test results revealed that all items contributed to the differentiation of the final clusters [111].

Table 5 presents the four segments of the Greek Generation Z cohort, which were extracted based on their behavior and attitudes towards entomophagy. It also displays the final cluster centers (FCC), the number of Zers per cluster (N), its percentage of the total sample, and the ANOVA tests (F values, sig.). The FCC were calculated as the mean of all variables within the final cluster, and reveals the behavioral characteristics of the typical subject for each cluster [112].

Chi-square and cross-tabulation tests were used to explore the socioeconomic and demographic characteristics of the Z cohort per segment (Table 6). Additionally, the chi-square test revealed that in all cases, the socioeconomic and demographic characteristics of the Z cohort did not produce statistically significant differences.

The extracted segments, their behavior and composition are as follows.

Segment No.1: The “Future potential insect consumer”, comprising 29.1% of the sample. This segment is overall the neutral segment in the sense that their FCC is distributed around the 4-point mark (neither agree nor disagree/neither likely nor unlikely) of the Likert and Likert type scale (3.57 < FCC < 4.50). This segment has the highest intention (FCC = 3.80) to consume edible insects or insect-based foods compared to the other segments (they tend to be neither likely nor unlikely to engage in entomophagy). The main drawback for not engaging in entomophagy seems to be “barriers to entomophagy (extrinsic and perceived intrinsic cues)”, since this group expose the highest FCC (FCC = 4.46) towards this dimension.

The profile of this segment is as follows. It is mainly composed of men (54.5%), the youngest members of the Greek Z cohort, i.e., 18–20 years old (44.4%), and single (96.5%). As the age group increases, its representation in the cluster decreases. Moreover, the members of this group in the majority have secondary education (64.6%), and in respect to occupation, most of them are university students (65.7%). Lastly, they live in the city (74.2%) and have a family net monthly income (FNMI) of up to EUR 600 (49.5%). Compared to the other segments, it has the highest proportion of university graduates and the lowest proportion of postgraduates. It also has the lowest percentage of businessmen and workers, and the highest percentage of dependents on others. In reference to income, it is the lowest income segment, as it has the highest percentage of members with an income of up to EUR 600, while 75.6% have an income of up to EUR 1000 (the lowest of all the groups compared). Lastly, it has the lowest percentage of members with an income of >EUR 2000.01 per month. 

Segment No. 2: The “Rejecters”, amounting to 26.7% of the sample. Its members show the following behavior and attitudes. They cannot be classified as food neophobic since their FCC = 3.50. They agree that eating insects is disgusting and that extrinsic and perceived intrinsic cues (insect appearance, taste, texture) are barriers to entomophagy. They also to consider that insect consumption likely poses a risk to human health. They somewhat disagree that (a) there is a food control conspiracy behind the future consumption of insects, (b) that they would rather starve than eat insects, and (c) that they could have consumed insects without knowing. Referring to intention to consume, they have the lowest FCC among all segments, and they totally disagree. Compared to the other segments, this segment (as well as the third segment) shows the highest disgust for insect consumption, and also recognizes the extrinsic and perceived intrinsic cues as barriers to insect consumption. In addition, both segments have the least intention to engage in entomophagy.

The socioeconomic and demographic characteristics of this segment are as follows. Males and single subjects are overrepresented (63.4% and 94.0%, respectively), and the youngest members of the Greek Z cohort are slightly overrepresented (38.9%). In addition, the other two age groups (i.e., 21–23 & 24–27) are equally represented. They have secondary education (62.5%), and concerning occupation, the majority are university students (62.0%). Finally, they live in the city (70.4%) and have a FNMI of up to EUR 600 (37.5%).

Compared to the other segments, it has the largest percentage of males, married, divorced, or widowed, and of university graduates and postgraduates, making it the highest educated group (26.4% having a graduate or postgraduate degree). Compared to the other clusters it has the smallest percentage of females, single subjects, postsecondary education, and dependents on others. 

Segment No. 3: The “Disgusted, prefer to starve”, representing 22.2% of the sample. Members of this segment exhibit the following behavior and attitudes. They marginally tend to be food neophobic since their FCC = 4.52 (>4.50). They also tend to totally agree that insect consumption is disgusting and that they would rather starve than eat insects. Besides that, they agree that extrinsic and perceived intrinsic cues are barriers to entomophagy. Additionally, they consider that entomophagy is somewhat likely to pose a risk to human health, and that there is a food control conspiracy behind the future consumption of insects. They tend to neither agree nor disagree that they have previously consumed insects without knowing it. Lastly, they have no intention of engaging in entomophagy. This segment is similar to the “Rejecters” segment, but exhibits stronger negative feelings of disgust, barriers to consumption, and negative attitudes. It is the group that prefers to starve rather than engage in entomophagy.

In this segment, male subjects (55.2%), the youngest Z cohort members, and singles (98.2%) are overrepresented. Furthermore, as the age group increases, its representation in the cluster decreases. Moreover, this segment has secondary education (67.5%), and in terms of occupation, the majority of them are university students (60.1%). Lastly, they live in the city (77.9%) and have a FNMI of up to EUR 600 (45.4%). 

Compared to the other segments, it has the highest proportion of single people, members living in the city and families with an income of > EUR 2000 per month. On the other hand, it has the lowest proportion of older members of the Z cohort (24–27 years old), married, divorced, or widowed, university graduates, and participants living in towns or villages. Finally, it has the smallest proportion of FNMI of EUR 600.01–1000.00 and EUR 1000.01–2000.00.

Segment No.4: The “Inconsistent”. This segment comprises 22.0% of the sample and its members are inconsistent in the sense that compared to the other segments, they disclose inconsistent to academic findings behavior. As noted previously, the main barriers to entomophagy recognized in academic research are food neophobia, disgust, and perceived health risk. This segment, on one hand, has the lowest FCC on most of the recognized variables to entomophagy (2.10 < FCC < 2.84), disagreeing or somewhat disagreeing with, but on the other hand, they also have no intention to consume insects (FCC = 2.29). Of the eight dimensions, only one has FCC > 3.00 (Perceived Health Risk; FCC = 3.69), meaning that they tend to consider that entomophagy is neither likely nor unlikely to pose a risk to human health. They are not food neophobic, nor do they exhibit disgust behavior. They also do not believe that there is a food control conspiracy, that extrinsic and perceived intrinsic cues are barriers to entomophagy, nor that they have consumed insects or insect-based foods unknowingly. Still, they do not have any intention to engage in entomophagy. In their case, other factors that have not been measured in this study may be the reason for low intention to engage in entomophagy, such as sociocultural values, personal beliefs, and social norms.

This segment is equally represented by males and females and is overrepresented by the youngest members of the Z cohort (49.1%), and single subjects (95.2%). They have secondary education (61.2%), and in terms of occupation, most of them are university students (55.8%). Lastly, the majority lives in the city (66.0%) and have a FNMI of up to EUR 600 (44.2%). 

Compared to the other segments, it has the highest percentage of the youngest Z cohort members, Zers with postsecondary education, employees (public/private) and businesspeople. Compared to the other groups, this group incorporate the highest percentage of people residing in towns and villages, and have a FNMI of EUR 600.01–1000.00. On the other hand, it has the lowest percentage of members of the Z cohort who are 21–24 years old, university students, and Zers residing in cities.

## 4. Discussion

The results of this study demonstrate that consuming insects is not broadly known among the members of the Greek Z cohort, as only 41.4% state that they are familiar with what it is (RQ1). This percentage is smaller than the percentage found in other studies. For example, Verbeke [44], in his study referring to consumers in Belgium, found that 71.5% of the participants “indicated that they had heard about the eating of insects and knew what it meant”. Hénault-Ethier et al. [113] in their study of French Quebeckers found that 96.0% of the sample had knowledge of entomophagy, while Wilkinson et al. [114] in their study of Australian consumers found that 68.0% of their sample were aware of entomophagy. 

The small percentage of the sample that is aware of entomophagy can be attributed to the lack of association due to sociocultural nutritional issues. For example, the Z cohort continues to live with their family as a consequence of household income decrease and job losses (economic crisis and covid crisis). Greek families have ingrained in their culture, as in other Mediterranean countries, the scheme of the Mediterranean diet, which is still prevalent today, even though there is a major shift towards the Western civilization diet. This is evident from the responses of the members of Generation Z cohort who stated that 45.0% follow the Mediterranean diet. Lastly, the ingredients used in cooking (e.g., the type of cheese or olive oil) as well as cooking techniques are passed down from one generation to another. Since the Mediterranean diet does not incorporate entomophagy, it is logical for this cohort to lack familiarity.

With respect to prior insect consumption experience (RQ2), no participant reported prior consumption of insect or insect-based foods and drinks. Lorini et al. [115] in their study in Italy found that 7.8% of the sample had prior experience in entomophagy, while Lammers et al. [49] found that in their sample (N = 516, Germany), 12.4% had prior experience in insect-based food consumption. Ribeiro et al. [116] found that past experience with insect consumption was much higher in Norway compared to Portugal, and both higher compared to the findings of the current study. In addition, other studies in Europe found that only a small proportion of their sample practiced entomophagy [93,117]. On the other hand, Woolf et al. [103] in their study in the USA found that 25.9% of the participants, and Hénault-Ethier et al. [113], found that 68.0% of their sample (French Quebeckers) had previous experience in insect-based food consumption. 

Concerning the insect-based food and drinks that the participants are willing to try (RQ3), in the majority, the ones that have a chance in the future to be consumed, are those where the insects are not visible. Therefore, insect-based flour or food with insect-based flour are more probable to be consumed. 

Regarding the question of whether participants are willing to replace animal protein with insect protein (RQ4), this study found that the vast majority (88.5%) are not willing to do so. This is in line with Vanhonacker et al. [118] who found that participants (N = 221 Flemish consumers) were not willing to replace animal protein with alternative proteins in their meals. These findings are also in line with previous findings regarding the Generation Z cohort for Greece [80,92]. In the present study, the 11.5% that is “willing” to replace animal protein with insect protein does not appear to be that high in reality. This conclusion arises from the fact that they were not willing to participate in sensory testing of insect-based foods, thus making the “first step” towards animal protein replacement.

As to interest in obtaining further information regarding entomophagy (RQ5), 20.4% answered yes, implying that only one-fifth of the sample is “open” to information regarding entomophagy. Therefore, for the one-fifth of the sample, there is a chance that gaining knowledge about the nutritional value of edible insects in the future could reduce negative attitudes towards insect-based food and drink consumption. Gere et al. [119] in their study (N = 400; Hungary) concluded that informing consumers about entomophagy and insect-based foods increased the likelihood that they would consume them. Likewise, in this study, a very small percentage (6.3%; N = 47), reported that they were interested in participating in sensory tests (RQ5), although when contact information was requested, only one was factual. This reveals that while intention may be high (tending to be moderate in our case), actual behavior is low. Previous researchers have identified the gap between intention and behavior [120,121], while Sheeran [122] states “A meta-analysis of meta-analyses indicated that intentions explain 28% of the variance, on average, in future behavior”.

Segmentation analysis (RQ6) incorporated eight variables, i.e., “Food neophobia”, “Barriers (extrinsic and intrinsic cues)”, “Disgust” “Perceived health risks”, “Conspiracy theories”, “Consumption without knowing”, “Starvation than entomophagy”, and “Intention to consume/try”. The four segments that were identified were “Future potential insect consumer”, the “Rejecters”, the “Disgusted, prefer to starve”, and the “Inconsistent”.

The segments from this study cannot be directly compared to other studies since the basis of segmentation was different. For example, Niva and Vainio [93] segmented 1000 consumers in Finland based on past changes and future intentions in the consumption of beef, plant, and insect-based proteins. Ribeiro et al. [116], grouped participants based on the degree of acceptance of different forms of entomophagy. Others incorporated one or some of the variables used in this study to identify segments. For example, Brunner & Nuttavuthisit [94] grouped consumers in Switzerland (N = 542) and Thailand (N = 500) based on attitudes towards e.g., food neophobia, liking towards specific food, general health interest and willingness to engage in entomophagy. They identified four segments: early adopters, early majority, late majority, laggards. 

Therefore, any comparison of this study’s segments is made with caution and on a general basis (for example, based on the intention to engage without considering the variables tested). 

Observing all segments of this study, two things can be concluded. First that no segment is at the time being ready to engage in entomophagy, since the first group which exhibits the highest intention (FCC = 3.80), tends to neither likely nor unlikely engage in entomophagy. This is in contrast with most of the previous segmentation studies, which found at least one segment ready to adopt entomophagy in their diet [94,123,124]. Additionally, the findings of this study are in line with almost all previous researchers, who found segments that are reluctant to engage in entomophagy [124,125].

Secondly, it can be concluded that the Greek Z cohort cannot be categorized as food neophobic, and therefore, refraining from entomophagy is not related to this dimension. The results of this study are not consistent with most of the previously mentioned studies that included food neophobia, since it does not appear to be an indicator of entomophagy rejection for the Greek Z generation cohort.

Two segments, the “Rejecters” and the “Disgusted, prefer to starve”, illustrate the highest degree of disgust for insect consumption. This finding is in line with the findings of Sogari et al. [41], who found that disgust is a stronger indicator of acceptance or rejection of entomophagy than food neophobia. These two segments also consider the extrinsic and the perceived intrinsic cues as barriers to insect consumption, and the perceived health risk from entomophagy is also the highest among the groups. Perone [126] points out that visual cues can elicit strong feelings of disgust, but for edible insects, pathogen disgust appears to be equally important [54]. These two groups also exhibit the least intention to engage in entomophagy. Therefore, our findings are accordingly in line with the findings of the above academic literature (regarding disgust and intention to proceed to insect consumption). This study’s findings are also partially in line with the results of Giotis and Drichoutis [95] research, referring to Greek consumers, since both studies found that acceptance of insect-based foods is low.

As for the rest of the Z cohort consumer groups, the first segment “Future potential insect consumer” is the only one that has the highest intention score to engage in entomophagy (FCC = 3.80), tending to the “neither likely nor unlikely” point of the 7-point Likert type scale. For this segment, the main barriers are extrinsic and perceived intrinsic cues, i.e., appearance, texture, and perceived taste. While this segment consists of 216 members of the Greek Z cohort, they were reluctant to participate in sensory tests, likely due to the above-mentioned barriers. 

Regarding the last segment, the “Inconsistent”, as mentioned earlier, there are probably other variables that influence their non-intention to engage in entomophagy, such as sociocultural aspects. Consumer attitudes towards trying or avoiding a new food derived from insects vary according to the cultural background [48]. For example, a study by Hartmann et al. [45] comparing Chinese and German consumers found that taste, nutritional value, familiarity, and social acceptance were ranked higher by the Chinese. Another study by Brunner and Nuttavuthisit [94] comparing Swiss and Thai consumers found that consumers in countries with an entomophagy tradition behaved quite differently than those without.

### 4.1. Theoretical Implications

This study has noticeable theoretical implications and fills the gaps in the existing literature on entomophagy regarding generational cohort behavior and attitudes. Most of the consumer-oriented research on entomophagy neglects the generational cohort theory; therefore, this study contributes to the current literature. Likewise, existing knowledge about the Z generation cohorts’ behavior, attitudes, and characteristics related to entomophagy is extremely lacking, and this study’s findings offer insight into all, especially for the reasons that it is rejected by them. Additionally, it fills the gap in the extant literature regarding market segmentation towards entomophagy, as it focuses on the Z cohort whereas no previous study was recognized.

Lastly, regarding the eight variables for the segmentation analysis, the attitudinal items included, originated from the factual statements of the Generation Z cohort participating in the qualitative research. Factor analysis extracted the three attitudinal dimensions, namely “Conspiracy theories”, “Consumption without knowledge”, and “Starvation than entomophagy”, which have not been previously identified in the existing quantitative literature (Sogari et al. [47] found conspiracy theory in their qualitative study) as a rejection of entomophagy and thus, can be used in future research.

### 4.2. Marketing Implications

The findings of this study provide noteworthy managerial directions. First of all, it reveals that the introduction/ market entry of insect-based food products in the Greek market targeting the Z cohort would probably fail at the current time. Therefore, the following presented directions will lead to fruitful outcomes in the long run.

In order for Z cohort members to substitute meat proteins for other proteins, it is critical to encourage them to consume food in a sustainable manner, thereby raising awareness of sustainability, i.e., increasing the number of “Green Zers” [127]. Therefore, key points for governments, green organizations as well as companies working on sustainability issues, sustainable food consumption, and alternatives to animal protein foods are to provide educational pathways and sources for the potential consumer, and to deliver information that the consumer trusts about the product.

Meat consumption has many health and environmental drawbacks; therefore, it is suggested that humanity should turn to alternatives to meat proteins. Currently, the Z cohort seems to need to be educated on sustainability issues, especially on meat protein substitution. Generation Z is a high-tech cohort and is looking for non-traditional educational avenues, and would be intrigued by a pedagogical style that would offer them memorable experiential quality and value. Therefore, gamification learning can be implemented in early school years [128,129], in order to achieve long-term results.

The most prominent novel alternative to meat proteins are plant-based meat analogues, insect-based foods, cultured meat, and algae [62]. Generation Z cohort consumers need to be educated about these alternatives to meat in respect to the benefits to their health and the sustainability of the planet. In many parts of the world, technological advances are quite popular in the early grades of school, even at elementary school level [130,131]. On the other hand, these practices are emerging in Greece, and are considered a breakthrough especially for environmental education [132,133].

This research revealed low self-reported familiarity with insect consumption. The starting point for marketing insect-based foods and drinks is to provide awareness. Therefore, informative communication campaigns need to be executed in Greece to enlighten the Z cohort consumers about what entomophagy is and the high nutritional value of insects (high protein and fat content, essential amino acids, minerals, and vitamins). A digital marketing communication campaign pointing out entomophagy’s low environmental impact, and what type of edible insects and insect-based foods are available, is also required. Additionally, the Generation Z cohort needs to be assured about the safety aspects of consuming these products, an example being potential allergic reactions. 

Since entomophagy is not part of modern Greek culture (and insect-based foods are not currently available on the market, to the authors’ knowledge), marketing campaigns will take time to achieve positive results. In view of the fact that members of Generation Z are “born in technology”, enjoy innovation, and are experience-motivated, these campaigns should be reinforced through other means, such as high-tech interactive approaches. Corporate websites could include links to videos, virtual reality (VR), augmented reality (AR), and mixed reality experiences with insect-based foods or beverage preparations. Sensory science has advanced significantly in recent years using VR and AR, and is rapidly becoming a key tool for predicting the success of foods in the marketplace [134].

For example, the combined use of augmented reality and “bug banquets” could motivate members of the Z cohort, even out of curiosity, to try insect-based food or drinks. Looy and Wood [135] [p.37] presented “bug banquets” to middle school, high school, and university students, and found that “they may help prepare people to respond more positively in future encounters with these species”. In addition, insect-based products could be marketed with the slogan “novel experiences with exotic foods” or “exotic experiences with novel foods” to capitalize on this cohort’s experiential motivation.

Moreover, gamification could be used to promote entomophagy, especially among younger members of the Z cohort. For example, Jones et al. [129] used an interactive game with heroic characters within a fictional story, read by teachers to promote fruit and vegetable consumption in school.

Virtual tours regarding insect-based food safety control, food production, food preparation, food packaging and handling would positively influence the Z cohort’s behavior and reduce their perceived high health risk from insect-based food consumption. 

Influencers are also an emerging phenomenon in Greece, affecting the behavior of the Z generation cohort members, and could be contacted by insect-based food companies. Instagram is a very powerful visual tool, and can be utilized to showcase different insect-based dishes or dishes prepared with insect-based ingredients. Alongside, Facebook sharing of posts and photos combined with prizes could raise the interest of this cohort in seeking information about entomophagy. Statista’s data [136] indicates that in Greece, 4.2% of individuals aged 13–17 years old and 24.1% of individuals aged 18–24 years old use Instagram, while these percentages were 3.4% and 17.8%, respectively, for Facebook users (data for July 2021).

Disgust seems to be the biggest issue, as well as perceived extrinsic and intrinsic cues for rejecting entomophagy. This finding, in conjunction with the insect-based food and drinks that this cohort is willing to try, indicates that the products that should be marketed, are those where the insects are not visible, such as insect-based flour, chips, or energy drinks. That being the case, free samples of energy drinks could be offered at “neuralgic” points of the Z cohorts’ interaction, such as at the SCHOOLWAVE festival and the ROCKWAVE festival. Lastly, segmentation of the Z cohort revealed that low-income Zers are the ones most likely to engage in entomophagy in the future. Therefore, insect-based protein products could be targeted to low-income families, as a safe substitute for meat protein products.

Overall, as mentioned earlier, these measures will bear fruit in the long term, as entomophagy is not “seriously” considered by participants in the short term.

## 5. Conclusions, Limitations, and Directions for Future Research

This study suggests that the Greek Z cohort is reluctant to engage in entomophagy, since three out of four segments interpreting 70.9% of the sample consider it unlikely or completely unlikely to engage in insect-based food consumption. Moreover, the only future potential segment is currently tending to be neither likely nor unlikely to engage in entomophagy. 

Based on eight behavioral and attitudinal dimensions, four characteristic segments of the Z cohort are identified: the “Future potential insect consumer” (29.1%), the “Rejecters” (26.7%), the “Disgusted, prefer to starve” (22.2%), and the “Inconsistent” (22.0%). From these segments, it appears that the Greek Z generational cohort is overall not food neophobic and thus, not engaging in entomophagy is not related to it. Disgust, extrinsic and intrinsic cues, and negative attitudes are the main barriers to engage in entomophagy for them.

This study is exploratory in nature, and even though it contributes to the existing literature on entomophagy and has provided novel information in academia with reference to the Z generation cohort’s behavior and attitudes, it possesses some unavoidable limitations. Further conclusive explanatory research is needed to confirm these findings. Second, this study focuses on one generational cohort, therefore extending the research to the other cohorts of the country would be of interest. Third, a non-probability sampling method was used; thus, generalization of the findings is not applicable. The fourth limitation is that the research was designed for one country, Greece, and therefore generalizations to other Mediterranean or European countries cannot be done. The last limitation considers other variables that influence entomophagy but are not included in the study, such as socio-cultural issues (e.g., religion or traditional meals) and social norms. These limitations may provide point by point avenues for future research on the issue, not only for the Greek context but also studying other countries and cultural contexts. In addition, the eight variables incorporated in the study for segmentation purposes can be adopted by other researchers to validate our findings, and to explore individual (and not only cohort) behavior and attitudes towards entomophagy. Beyond these limitations, this study is considered very important, since it provides extensive insight into the behavior and attitudes of the youngest adult generational cohort for which marketers lack information, and thus signifies future trends in the Greek entomophagy market.

## Figures and Tables

**Table 1:** Questions used in the study, the authors from whom they were taken, and the scale used.

Questions/Issues of the Research	Items in the Question	Adopted from	Scale Used
Awareness of entomophagy	3	Verbeke [44]	Multiple choice (one choice)
Previous consumption experience	1	Sogari et al. [41]	Yes-no dichotomous answer
Willingness to consume specific edible insects or insect-based food/drinks	22	Ribeiro et al. [102]; Woolf et al. [103]; Hartmann et al. [45]	Yes-no dichotomous answer
Willingness to substitute animal protein with insect protein	1	Verbeke [44] (modified)	Yes-no dichotomous answer
Interest to learn more about entomophagy	1	Authors	Yes-no dichotomous answer
Interest in participating in sensory tests	1	Authors	Yes-no dichotomous answer
If interested, insert contact information	1	Authors	Open-ended question
Food Neophobia	10	Pliner et al. [104]	7-point Likert Scale
Barriers to entomophagy (extrinsic and perceived intrinsic cues: appearance, taste, texture)	3	Cicatiello et al. [42]	7-point Likert Scale *
Disgust towards insect consumption	10	La Barbera et al. [105] & Ribiero et al. [102]	7-point Likert Scale
Intention (direct)	3	La Barbera et al. [105]	7-point Likert Scale
Perceived risk for human health	1	La Barbera et al. [105]	7-point Likert-type Scale
Attitude	12	Qualitative research	7-point Likert Scale

**Table 2:** Profile of participants in the field research.

Sample Characteristics	Frequencies	Percentages (%)
Gender		
Male	418	56.3
Female	324	43.7
Generation Z Cohort		
18–20	323	43.6
21–23	217	29.2
24–27	202	27.2
Marital status		
Single	711	95.8
Married/Divorced/Widowed	31	4.2
Education		
Secondary	474	63.9
Postsecondary	99	13.3
Graduate	121	16.3
Postgraduate	48	6.5
Profession		
Employee (public, private)	135	18.2
Businessman	42	5.7
Worker	25	3.4
University student	454	61.1
Dependent (housekeeper, unemployed)	86	11.6
Area of residence		
City	535	72.1
Town	100	13.5
Village	107	14.4
Net Monthly Family Income (in euros)		
≤600.00	326	43.9
600.01–1000.00	193	26.0
1000.01–2000.00	122	16.4
≥2000.01	101	13.7

Source: The authors.

**Table 3:** Willingness to try insect and insect-based food/drink.

Willingness to Try Insect and Insect-Based Food/Drink	Yes		No		Total
	N	%	N	%	
Bakery products containing insect flour	152	20.5	590	79.5	742 (100%)
Rice/pasta enriched with insect flour	145	19.5	597	80.5	
Fried/grilled/toasted whole insect	144	19.4	598	80.6	
Cookies based on cricket flour	142	19.1	600	80.9	
Protein bars containing insect protein isolate	140	18.9	602	81.1	
Chocolate chip cookies based on cricket flour	133	17.9	609	82.1	
Ground insects in sauces/chutneys	127	17.1	615	82.9	
Ground insects in burgers/meatballs/nuggets	126	17.0	616	83.0	
A drink containing silkworm protein	122	16.4	620	83.6	
Sugar/chocolate covered insect	109	14.7	633	85.3	
Deep-fried crickets	104	14.0	638	86.0	
Ants	104	14.0	638	86.0	
Grasshoppers	103	13.9	639	86.1	
Cicadas	89	12.0	653	88.0	
Deep-fried silkworms	86	11.6	656	88.4	
Spiders and scorpions	85	11.5	657	88.5	
Mealworm	79	10.6	663	89.4	
Worms	78	10.5	664	89.5	
Crickets	75	10.1	667	89.9	
Caterpillars	72	9.7	670	90.3	
Cockroaches	69	9.3	673	90.7	
Scarab	63	8.5	679	91.5	

Source: The authors.

**Table 4:** Constructs and Reliability of scale.

Question/Construct	Total Variance Explained (%)	No. of Constructs after FA (Items per Construct)	Reliability of Scale Total (& Construct)
Food Neophobia (KMO = 0.948; BTS = 7,557,641; df = 66; *p* = 0.000)	65.5	1 (10)	0.952
Barriers to entomophagy (extrinsic and perceived intrinsic cues: appearance, taste, texture) (KMO = 0.682; BTS = 1,304,743; df = 3; *p* = 0.000)	79.3	1 (3)	0.753
Disgust towards insect consumption (KMO = 0.950; BTS = 9,653,682; df = 45; *p* = 0.000)	79.7	1 (10)	0.971
Intention (direct) to consume/try (KMO = 0.770; BTS = 2,050,811; df = 3; *p* = 0.000)	89.8	1 (3)	0.943
Attitudes towards insect consumption (KMO = 0.861; BTS = 5,422,242; df = 55; *p* = 0.000)	76.0 (31.6; 26.2; 18.2)	3 (5, 4 & 2; one was dropped)	0.900 (0.899; 0.858 & 0.945)

Source: The authors; KMO=Kaiser-Meyer-Olkin Test for Sampling Adequacy; BTS=Bartlett’s test of sphericity; df=degrees of freedom

**Table 5:** Segments of the Z cohort based on barriers of entomophagy.

Segmentation Variables	Final Cluster Centers				ANOVA	
	Clusters					
	1st N = 216 (29.1%)	2nd N = 198 (26.7%)	3rd N = 165 (22.2%)	4th N = 163 (22.0%)	F	Sig.
Food neophobia	3.62	3.50	4.52	2.65	49.520	0.000
Disgust	4.21	6.19	6.61	2.84	457.154	0.000
Barriers to entomophagy (extrinsic and perceived intrinsic cues)	4.46	6.32	6.43	2.58	464.883	0.000
Perceived health risk	3.97	4.71	5.02	3.69	31.959	0.000
Conspiracy theories	4.26	3.47	5.00	2.39	128.721	0.000
Consumption without knowing	4.25	2.89	3.82	2.34	82.187	0.000
Starvation than entomophagy	3.57	3.17	6.52	2.10	350.147	0.000
Intention to consume	3.80	1.31	1.37	2.29	237.300	0.000

Source: The authors.

**Table 6:** Profile of University students’ Generation Z cohort segments.

Cluster Profile	Cluster I	Cluster II	Cluster III	Cluster IV
	Percentages (%)			
Gender (x2 = 7.197; df = 3, *p* = 0.068)				
Male	54.5	63.4	55.2	50.3
Female	45.5	36.6	44.8	49.7
Age groups (x2 = 9.024; df = 6, *p* = 0.174)				
18–20	44.4	38.9	42.9	49.1
21–23	30.3	30.6	33.8	21.8
24–27	25.3	30.5	23.3	29.1
Marital status (x2 = 4.442; df = 3, *p* = 0.218)				
Single	96.5	94.0	98.2	95.2
Married/divorced /widowed	3.5	6.0	1.8	4.8
Education (x2 = 9.395; df = 9, *p* = 0.402)				
Secondary	64.6	62.5	67.5	61.2
Postsecondary	12.1	11.1	14.7	16.5
Graduate	18.7	17.1	12.3	16.4
Postgraduate	4.6	9.3	5.5	6.1
Profession (x2 = 14.288; df = 12, *p* = 0.283)				
Employee (public-private)	16.7	18.5	16.6	21.2
Businessman	2.5	7.0	6.1	7.3
Worker	1.5	4.2	4.9	3.0
University student	65.7	62.0	60.1	55.8
Dependent (housekeeper, unemployed)	13.6	8.3	12.3	12.7
Area of residence (x2 = 6.975; df = 6, *p* = 0.319)				
City	74.2	70.4	77.9	66.0
Town	13.1	13.4	10.4	17.0
Village	12.7	16.2	11.7	17.0
Net Monthly Family Income (in euros) (x2 = 16.303; df = 9, *p* = 0.064)				
≤600.00	49.5	37.5	45.4	44.2
600.01–1000.00	26.3	27.8	21.5	27.9
1000.01–2000.00	14.6	19.4	13.5	17.6
≥2000.01	9.6	15.3	19.6	10.3

Source: The authors.
